# Platelet function in the postprandial period

**DOI:** 10.1186/1477-9560-10-19

**Published:** 2012-09-03

**Authors:** Helmut Sinzinger, Robert Berent

**Affiliations:** 1 Institute for Diagnosis and Treatment of Lipid Disorders and Atherosclerosis (ATHOS), Vienna, Austria; 2 Wilhelm Auerswald Atherosclerosis Research Group (ASF), Vienna, Austria; 3 Department of Nuclear Medicine, Medical University of Vienna, Vienna, Austria; 4 Center for Cardiovascular Rehabilitation, Rehabilitationszentrum Austria, Bad Schallerbach, Austria

**Keywords:** Postprandial hyperlipidemia, Platelet activity, Platelet aggregation, Platelet count, Atherosclerosis

## Abstract

**Background:**

Postprandial hyperlipidemia and hyperglycemia have been related to cardiovascular events. Among different underlying mechanisms platelet activation seems to be responsible too. No comparable data between various tests in normo- vs. hyperlipidemics before and at different time intervals are available after a fat meal. We aimed to compare 9 of them within the same patients at several time points in postprandial hyperlipidemia.

**Results:**

For some tests baseline values between the groups were significantly different (TXB_2_, platelet sensitivity, sedimentation and WU-test). However, hyperlipidemia revealed a variable influence on the tests examined. Some of the available tests apparently sensitive to show platelet activation reflect the increase in triglycerides (TG), such as the sedimentation index. ADP-induced platelet aggregatory activity in count adjusted washed isolated platelet samples during postprandial hyperlipidemia indicates mildly enhanced platelet activity, but does not seem to induce significant changes in aggregation. In patients with severe hypertriglyceridemia (> 400 mg/dl fasting) changes in platelet function are more pronounced due to delayed decay and may last up to 16 hours paralleling TG reaching the prevalue. The overwhelming majority of platelet function tests do not significantly respond to postprandial hyperlipidemia. The correlation between the tests applied is poor. For standardization purpose, platelet aggregation tests, aimed to examine proaggregatory capacity in atherosclerosis, should only be performed at the same time of the day after a fasting period > 6 hours. The great variation in preanalytical work-up on comparison of various tests, large number of platelet tests available and their respective potential value are discussed.

**Conclusions:**

At present, the suspicion that platelet function is significantly activated in the postprandial period cannot be supported by any of the tests used. The information provided is valuable to know for which test and group of patients a fasting period of which duration is recommendable.

## Introduction

Long-term high intake of saturated fats is responsible for the morbidity and mortality in coronary heart disease. Postprandial (but not fasting) triglycerides [1] and small chylomicron remnants [2] were related to coronary heart disease and the progression of coronary atherosclerosis, respectively. Along this line, postprandial angina after a fat meal has been recognized for many centuries as a marker of severe coronary artery disease [3]. Moreover, a variety of studies are claiming an activation of platelets and plasmatic coagulation in the postprandial period [4,5]. Although the extent of postprandial (hyper-)lipidemia has been reported to imbalance hemostasis, the significance of postprandial lipid concentration, fatty acid metabolism and platelet function is still under debate. Repetitive activation of platelets could be responsible for initiation, development and progression of atherosclerosis and enhanced incidence of arterial thrombosis. While a variety of studies are available examining the response of various platelet function tests on differently induced postprandial stages, a comparative study running several tests in one particular patient after a standardized meal is not available.

A PubMed search performed on January 24^th^, 2012 using various key word combinations reveals a surprisingly small number of papers dealing with the issue of this contribution (Table 1).

**Table 1:** PubMed literature survey

**Key word**	**References found**
PP	16598
PP + PL	125
PP + PL function	121
PP + PL aggregation	48
PP L + PL	79
PP L + PL function	79
PP L + PL aggregation	33
PP G + PL	38
PP G + PL function	37
PP G + PL aggregation	14
PP G + L + PL	16
PP G + L + PL function	16
PP G + L + PL aggregation	6

PP … postprandial, PL … platelet, L … lipids, G … glucose.

## Patients and methods

Blood for platelet function testing was always drawn during 7.30 a.m. and 9.00 a.m. in the morning after overnight fasting (more than 16 hours) from a non-occluded cubital vein. Before blood withdrawal patients were allowed to rest for 30 minutes. For platelet function testing special care was taken in order to avoid ex-vivo and in-vitro activation. Platelet count was adjusted in all aggregation test samples to 250x10^3^/μl. Platelet function tests performed were the determination of platelet proteins (ß-thromboglobulin, platelet factor 4) and plasma thromboxane B_2_ by means of a radioimmunoassay, as well as platelet sensitivity to PGI_2_ (ADP-induced aggregation), sedimentation index, WU-test for reversible circulating platelet aggregates, retention, migration and in-vitro radiolabeling of platelets.

Postprandial testing was performed after a meal consisting of 80 g fat in 2 rolls. Familial hypercholesterolemia was defined as isolated total cholesterol > 250 mg/dl without lipid lowering agents and severe familial hypertriglyceridemia (HTG) as fasting triglycerides (TG)-values above 400 mg/dl before treatment and after exclusion of secondary HTG. All participants were non-smokers. None of the patients was taking any drug since > 2 weeks before blood withdrawal.

### Statistical analysis

Values are presented as x ± SD, calculation for significance was done by Student’s t-test. A p < 0.01 is considered as significant.

## Results

The prevalues for thromboxane (TX) B_2_, the platelet sensitivity against prostaglandin (PG) I_2_, the WU-test and the sedimentation index (SI) show a difference between normolipemics vs. hypercholesterolemics (HC) and hyperlipidemics with metabolic syndrome (MS) (Table 2). All other parameters are not significantly different. Tests predominantly being based on the physical interaction between large lipoproteins and platelets, such as SI und WU-test, apparently are more pronounced in HTG while platelet sensitivity being a measure for the PGI_2_-surface receptors are more pronounced in hypercholesterolemia. The behaviour of the various tests after a fat meal is quite different. The trend towards an increase in peripheral platelet count is quite comparable in the 3 different groups of patients (Table 3) reaching only at 3 and 6 hours, respectively, statistical significance. A very severe increase can be noted for the SI being most pronounced in MS-patients. Interestingly, this response in MS-patients apparently correlating to the long-lasting increase in TG remains significant for > 12 hours, while in normolipemics and HC the increase is much less pronounced and by far shorter lasting. The reason seems to be a correlation between SI and TG (r = 0.7413; p < 0.001). All the other tests examined do not show any significant change after the fat load (Table 3).

**Table 2:** Prevalues of the platelet function parameters examined

**Parameter**	**NL**	**HC**	**MS**	**Units**
count	211 ± 10	216 ± 13	217 ± 11	x10^3^/μl
ßTG	51.7 ± 2.2	55.6 ± 2.3	52.3 ± 2.8	pg/ml
PF4	16.6 ± 2.4	18.9 ± 2.7	18.6 ± 2.5	pg/ml
TXB_2_	7.4 ± 1.3	12.8 ± 2.3*	10.7 ± 1.9*	pg/ml
PS PGI_2_	0.96 ± 0.09	1.82 ± 0.51*	1.65 ± 0.22*	ng/ml
SI	2.9 ± 0.4	4.1 ± 0.6*	6.8 ± 2.2*	%
WU	0.95 ± 0.02	0.86 ± 0.04*	0.78 ± 0.06*	%
retention	88.4 ± 3.2	87.1 ± 3.7	89.0 ± 3.8	%
migration	386 ± 21	397 ± 26	391 ± 22	mm^2^

values in x ± SD; n = 28 each group (14 m/14 f, aged 31 – 56 years); *) p < 0.01; NL indicates normolipemics; HC, hypercholesterolemics; MS, metabolic syndrome; PS, platelet sensitivity; count, platelet count; ßTG, ß-thrombogloblin; PF4, platelet factor 4; TXB_2_, thromboxane B_2_; PS PGI_2_, platelet sensitivity to prostaglandin I_2_; SI, sedimentation index; WU, WU-test.

Only TXB_2_, PS PGI_2_, SI and the WU-test reveal significant differences between NL vs. HC- and MS-patients, respectively. TXB_2_ and PS PGI_2_ seem to be more closely related to cholesterol, SI and the WU-test to TG.

**Table 3:** Influence of a fat meal on platelet function parameters

	**3 hours**	**6 hours**	**12 hours**	**group**
count	5.2 (3.3)	7.5 (2.2)*	3.1 (1.4)	NL
	4.9 (2.6)	8.3 (2.5)*	4.2. (1.5)	HC
	5.0 (2.4)	7.7 (1.8)*	2.9 (1.3)	MS
ßTG	1.5 (5.3)	2.1 (5.0)	1.0 (3.8)	NL
	2.1 (6.3)	3.0 (7.1)	2.8 (± 5.4)	HC
	0.7 (8.4)	1.3 (10.6)	- 0.3 (6.4)	MS
PF4	0.7 (5.3)	2.3 (2.8)	- 1.4 (5.2)	NL
	1.3 (4.8)	2.0 (4.2)	0.8 (6.3)	HC
	1.1. (3.6)	1.2 (5.7)	- 1.1 (5.4)	MS
TXB_2_	4.2 (12.2)	0.3 (6.3)	- 1.3 (3.8)	NL
	- 0.6 (8.5)	1.4 (5.4)	1.7 (6.5)	HC
	2.0 (9.1)	1.3 (7.5)	- 0.6 (7.1)	MS
PS PGI_2_	−1.3 (4.4)	- 0.5 (3.1)	- 0.2 (3.6)	NL
	1.0 (2.7)	0.6 (2.3)	0.7 (2.7)	HC
	- 0.8 (2.5)	0.9 (3.1)	1.3 (1.9)	MS
SI	28.7 (30.3)	2.1 (20.2)	3.6 (10.6)	NL
	21.4 (17.9)	0.6 (14.3)	2.0 (7.5)	HC
	84.6 (25.8)*	86.3 (21.6)*	26.8 (13.9)*	MS
WU	3.4 (4.6)	2.7 (2.4)	0.7 (3.4)	NL
	2.7 (3.3)	1.0 (3.1)	1.6 (2.6)	HC
	3.3 (3.3)	2.2 (3.7)	2.5 (4.1)	MS
retention	- 3.1 (6.3)	4.7 (7.4)	2.2 (3.9)	NL
	2.4 (5.9)	2.5 (5.8)	0.4 (6.2)	HC
	4.7 (6.1)	6.8 (6.0)	2.1 (4.5)	MS
migration	1.3 (3.2)	2.4 (3.4)	2.0 (3.20	NL
	2.8 (2.7)	2.4 (4.0)	1.7 (2.0)	HC
	2.2 (3.8)	2.0 (3.9)	1.1 (1.8)	MS

n = 12 each (6 m/6 f, age 30 – 48 years); values in % vs. prevalue (SD); *) p < 0.01; for abbreviations see Table 2.

A fat meal increases only peripheral platelet count (maximum at 6 hours) in all groups, SI only in MS-patients for up to 16 hours.

Measuring ADP-response of platelets in platelet rich plasma revealed a decreased response in the tangent of the response curve (data not shown) as well as the maximal amplitude (Table 4). In contrast, testing isolated washed platelets there is a trend towards an increase in aggregation response which is significant for HC after 3 hours and for MS up to 6 hours. Also the extent of the change is most pronounced in MS patients. In (normal weight) patients with severe familial HTG deviation of aggregation response to ADP is more pronounced and longer lasting as compared to HC- and even MS-patients (Table 5).

**Table 4:** ADP-response of washed platelets (WP) vs. in platelet-rich plasma (PRP)

	**3 hours**	**6 hours**	**12 hours**	**group**
WP	3.7 (7.1)	2.2 (5.4)	0.6 (4.1)	NL
	4.3 (3.3)*	1.4 (3.8)	- 0.2 (3.3)	HC
	15.3 (6.2)*	12.7 (6.0)*	4.7 (2.9)	MS
PRP	- 4.2 (5.3)	- 2.6 (2.4)	1.0 (1.6)	NL
	- 4.0 (3.9)	- 1.1 (2.3)	- 0.4 (0.9)	HC
	- 13.7 (5.9)*	- 11.6 (3.1)*	- 3.3 (1.5)	MS

n = 20 each (12 m/8 f, age 30 – 48 years); values in % change (SD) vs. prevalue; WP indicates washed platelets; PRP, platelet-rich plasma; for further abbreviations see Table 2.

Aggregation response is mainly influenced during the initial 3 hours after a meal in MS-patients. In NC and HC the alterations are small and short lasting. In WP there is an in part significant activation, while in PRP turbidity causes a decrease to a comparable extent.

**Table 5:** Platelet aggregation in washed platelets vs. platelet-rich plasma in severe familial hypertriglyceridemia

	**1 hour**	**3 hours**	**6 hours**	**12 hours**	**16 hours**	
A.S. 41, ♂	- 10.6	- 15.7	- 17.3	- 10.8	- 1.0	PRP
	13.4	18.9	20.3	11.4	2.3	WP
D.R. 37, ♀	- 9.2	- 13.7	- 15.4	−8.6	- 0.3	PRP
	12.0	16.2	17.3	10.3	0.4	WP
J.O. 29, ♂	- 7.6	- 12.2	- 12.1	- 7.6	- 0.9	PRP
	10.1	13.8	14.7	9.9	2.0	WP
H.M. 54, ♂	- 11.3	- 14.7	- 18.6	- 11.7	- 3.3	PRP
	13.0	17.2	21.1	13.0	4.1	WP
D.W. 44, ♂	- 8.6	- 9.3	- 12.9	- 5.4	- 1.6	PRP
	11.0	12.6	15.2	8.4	- 0.3	WP

values in % changes vs. prevalue; PRP indicates platelet-rich plasma; WP, washed platelets.

In severe HTG platelet function change induced by ADP is particularly long lasting up to 16 hours. Calculating the group statistics reveals that after 16 hours differences are no longer significant.

## Discussion

### Postprandial period

Platelet function testing in dyslipoproteinemias suggested that lysolecithin and phosphatidylinositol may be involved in modulating platelet response to aggregating agents [6]. Early reports showed that acute postprandial HTG causes moderate activation as examined in isolated platelets [7-9]. The platelet proteins ß-thromboglobulin (ßTG) and platelet factor 4 (PF4) were reported to be unaffected by a fat meal [10], while, in contrast, at the same time P-selectin expression was increased. Platelet microparticles exhibiting proatherogenic and procoagulant properties have been found to be increased in postprandial HTG showing a significant correlation to the actual TG level measured [11]. Although fat meals have shown acute effects on platelet function, apparently they are not mediated via the fatty acid composition of the acute meal [12]. Using isolated washed platelets derived from postprandial blood samples, Aviram’s group [13-15] in a broad set of experiments found that the intake of fat meals induces an acute disturbance in platelet aggregation. The changes are more pronounced in hyperlipemic patients and after a saturated fat meal. Nordoy et al. confirmed that, as an acute intake of saturated and polyunsaturated fatty acids increases platelet sensitivity to thrombin and collagen [8,9].

The methodological aspect is highlighted by a report [16,17] that the consumption of 100 g fat after a 12 hours fasting period causes a significant decrease (!!) in platelet aggregability upon both, ADP and collagen. The authors did not discover a correlation between the inhibition of platelet aggregation and the increase in TG during postprandial hyperlipidemia. This reduction in maximum platelet aggregation response during the postprandial stage (p < 0.001) was also described by Ahuja et al. [18]. At the same time there was no change in PG, including TXB_2_. However, in agreement with Aviram’s and Nordoy’s findings [8,9,13,14], using washed human platelets we were able to show that platelet activity is not significantly affected and the changes described are simply due to the turbidity of the plasma [19,20], a phenomenon usually seen when TG exceeding 400 mg/dl.

TX was not influenced, irrespective of whether the fat meal was taken together or without alcohol [21]. The interpretation of the data is further complicated by the fact that postprandial hyperinsulinemia stimulates platelet aggregation [22] which might particularly interfere in MS-patients, a large ever growing part of the population. On the contrary, in hyperinsulinemia a reduced platelet aggregation (ADP, collagen, epinephrine) was reported when euglycemia was maintained by clamping [23]. Westerbacka et al. described that normal in-vivo insulin action inhibits platelet interaction with collagen and aggregation with several agonists [24]. After a fat meal Wiens et al. found in healthy volunteers no change in platelet function tested by the platelet function analyzer (PFA-100) and circulating platelet aggregates, the so-called WU-test [25]. They note a postprandial increase in platelet count at 6 hours up to 8% from baseline which is in agreement with our findings (x = 7.5 – 8.3%). Using the PFA-100, Karepov reported that HTG significantly influences platelet response to acetylsalicylic acid (ASA) [26], a finding which has not been confirmed by any other test. Different platelet preparation with respect to fasting prior blood withdrawal for platelet function testing is responsible for conflicting results.

Assuming that tea is frequently consumed with a meal, testing postprandial platelet aggregation on ADP was not different [27] in presence vs. absence of tea consumption. The evening meal did not have an influence on shear-induced platelet aggregation and urinary TX excretion either [21]. Delgado-Lista et al. showed that long-term ingestion of saturated versus non-saturated and omega-3 fatty acids resulted in a comparable postprandial response while basal levels were different [28]. In an in-vitro approach, virgin olive oil was shown to reduce TXB_2_[29]. In 31 healthy subjects acute alimentary lipemia resulted in a decreased expression of CD40L on platelets and a reduced plasma level, suggesting an enhanced turnover [30].

GMP-140, an α-granule membrane protein, was shown to be positively (p < 0.01) correlated to total- (r = 0.647) as well as LDL-cholesterol (r = 0.833), but not to TG [31]. In contrast, ß-TG and PF4 also derived from α-granules apparently are not significantly elevated in HC. Flow cytometry with monoclonal antibodies showed that platelet P-selectin expression was significantly higher 2 ½ hours after a 40% fat meal consumption as compared to fasting values which were achieved again after 6 hours. In contrast, platelet monocyte aggregation increased up to 6 hours [5]. In these healthy normal-weight people TG increased by about 50% [18,32].

### Metabolic syndrome (MS)

An ever increasing number of people are suffering from overweight and adiposity-induced MS with elevated TG and low HDL-cholesterol. MS patients have been characterized by a proinflammatory stage and a positive correlation to platelet activity markers [33]. HTG itself has been linked with platelet activation. Very low-density lipoproteins (VLDL) and remnant lipoproteins are associated with MS. VLDL and remnant lipoproteins elevated in patients with MS have the capacity to activate platelets [34]. The increasing number of people with MS may reveal different TG kinetics, in some of them normalization after a single meal in some of them may last up to 16 hours [35]. In type II diabetics the enhanced postprandial platelet reactivity was not influenced by glibenclamide or repaglinide [36] suggesting a lipid mediated action.

### Other factors to be considered

#### Role of modification of lipoproteins

In hypertriglyceridemic subjects the rate of oxidative modification of lipoproteins (VLDL, LDL, HDL, Lp(a)) is greatly increased. Waist circumference has been associated with an augmented oxidation injury as evidenced by isoprostane (8-epi-PGF_2α_) measurement. MS-patients may also show other types of modification, for instance glycation, glycoxidation, or malonylation. Platelet maximal aggregation was reported to be significantly (p < 0.05 – 0.01) increased [37] after oxidative modification of the respective lipoprotein. In-vitro experiments have demonstrated that advanced glycation end products may enhance platelet activity assessed by a variety of tests several-fold [38]. Platelet NO-production was significantly related with body-mass index, waist circumference in HTG [39] and glycation of LDL [40]. However, platelet NO-sensitivity postprandially has been shown to be unchanged [35]. A reduced paraoxonase activity correlating with low HDL-levels in HTG indicates an impairment in enzymatic antioxidant activity [41].

#### Low HDL in diabetes mellitus (DM)

A major clinical problem with type 2 DM is the enhanced platelet reactivity resulting in a prothrombotic state. As a consequence, diabetic platelets have been claimed to be less sensitive to antithrombotic drugs [42]. However, antithrombotic treatment has little impact on the stability of vulnerable plaques and does not target the phenomenon of platelet hyperreactivity. Recently, Calkin et al. reported that administration of reconstituted HDL caused a reduction of platelet aggregation response in DM subjects by promoting cholesterol efflux from platelets with an increased cholesterol content [43]. These findings demonstrate that reconstituted HDL is highly effective at reversing the excessive accumulation of cholesterol in platelet membranes in patients with low HDL as in diabetics. Furthermore, attention should be focused on the functionality of HDL. HDL may act as an antioxidant for LDL while becoming oxidized itself loosing significant parts of its atheroprotective benefits.

#### Hypertriglyceridemia (HTG)

HTG has been associated with cardiovascular disease since the early 70ies [44]. HTG is known to enhance platelet activation [45]. One responsible mechanism is the pathway via the apoE content of VLDL particles [46] and their interaction with the platelet LDL receptors. Moreover, successful treatment of HTG with bezafibrate decreases platelet GP53 expression, but not P-selectin and fibrinogen binding in a double-blind placebo-controlled study [45].

Elevated levels of non-fasting/postprandial TG directly correlate with elevated remnant cholesterol VLDL and remnant lipoproteins have the capacity to activate platelets and are closely associated with atherosclerosis [47].

#### Methodological standardization

At present there is still no widely agreed definition of postprandial lipemia [48]. There is also conflicting information on blood collection and platelet processing regarding fasting and feeding states [18]. Our data clearly indicate that for platelet function testing standardization of blood withdrawal is needed. Fasting TG levels are necessary to be standardized, but non-fasting levels much better reflect the cardiovascular risk much better [44,49]. Therefore, a fasting sample for platelet function studies should be mandatory [18] unless the objective is to determine acute effects of drugs and/or food. No studies as to the type of hyperlipidemia (vs. normolipemia), time-dependency and the TG kinetics are available.

Aznar et al. confirmed this inhibitory effect of TG on platelet activation [50] while in presence of chylomicrons no modification in platelet aggregation was revealed, which is in accordance with our findings. A circadian rhythm of platelet activity has also been claimed to be responsible for the increased incidence of cardiovascular events early in the day [51-53]. Permanent hyperlipidemia causes a different fatty acid content [54], membrane composition [6,55] and membrane fluidity of platelets. This results in an altered functional and labeling behaviour [56]. In contrast, platelets taken during postprandial hyperlipidemia even in severe HTG do not exhibit a different labeling behaviour (Table 6). On the other hand, the dietary fatty ingestion may induce an altered sensitivity of platelets to aggregation inducing agents [57]. A long-term mono-unsaturated fatty acid rich diet as a matter of fact by influencing platelet membrane fatty acid composition [58] was shown to reduce platelet aggregation induced by ADP and arachidonic acid in healthy young people.

**Table 6:** **Influence of fasting on radiolabelling behaviour of platelets with **^**111**^**In-oxine**

	**NL**	**HC**	**MS**	**HTG**	
LE	90.6 ± 2.7	46.2 ± 3.8*	86.9 ± 2.9	88.8 ± 2.7	fasting
	89.7 ± 2.5	46.8 ± 4.3*	87.5 ± 2.2	88.4 ± 1.9	non-fasting
REC	68.2 ± 3.0	22.7 ± 5.2*	63.7 ± 5.8	67.5 ± 4.0	fasting

values in % (mean ± SD); n = 12 each subgroup (6 m/6f); *) p < 0.01 (vs. NL); LE indicates labelling efficiency; REC, recovery; for further abbreviations see Table 2.

Postprandial hyperlipidemia does not affect LE or REC in any of the patient groups.

Platelet lipoprotein binding has been claimed to be related to platelet reactivity. Patients with clinically manifested atherosclerosis, hypercholesterolemia and DM exhibit a significantly decreased high-affinity LDL- and HDL-binding on platelets [59,60] being associated with an increased platelet activity as demonstrated for TXB_2_, platelet proteins and ADP-induced platelet aggregation. This inverse relation has been found to be reversed with lipid lowering (statins, etofibrate, gemfibrozil, cholestyramine) treatment [61]. While the chronic effect of increased lipids and lipoproteins on platelet lipoprotein binding sites is well investigated, it is unknown whether there are acute changes during postprandial hyperlipidemia reflected at the platelet LDL- and/or HDL-receptor level.

Postprandial hyperglycemia in early type II diabetics was associated with an increased platelet activation as judged by the urinary excretion of the elevated 11-dehydro-TXB_2_-compound [62] and an activation of protein kinase C (α, ß_1_, ß_2_) in platelets, a potential candidate for mediating increased cardiovascular risk [63].

Only few data are available for most of the tests. The SI [64] defined as the percentage of platelets in platelet rich plasma (PRP) after centrifugation at 1600 g vs. PRP is the only one parameter which is found significantly increased after a fat meal known and correlates well with TG [65] apparently reflecting physical interaction of platelets with large lipoprotein particles.

The prevalues of various platelet function parameters reveal that the platelet sensitivity to PGI_2_ is the most abnormal parameter in HCNMS vs. NL. On the other hand, the SIN metabolic syndrome beside the Wu-test shows the most pronounced changes. A fat meal in all the 3 groups tested shows an increase in peripheral platelet count, while the sedimentation index is only significantly altered in MS-patients. Remarkably (Table 4), the platelet function testing reveals an opposite behaviour if done in washed cells vs. PRP, the elevated triglyceride rich lipoproteins being responsible for the change in the transmission. The individual follow-up monitoring up to 16 hours shows that there is a discrepancy in the results of platelet aggregation performed in washed platelets vs. those in PRP, which at 16 hours fasting almost disappears.

Interestingly, radiolabeling behaviour of platelet only significantly affected in patients with hypercholesterolemia, apparently due to the different membrane composition, fasting had no significant effect on labeling efficiency as on recovery.

As a matter of fact, all the data discussed in this paper were derived from non-smokers. While it is well known that the baseline values for most of the tests are differing between cigarette smokers and non-smokers, a comparative evaluation of these 2 groups on either a carbohydrate rich or a fat meal has not been performed. Other components such as obesity and hypertension may alter baseline platelet function. However, only in MS-patients the postprandial response seems to be influenced. Moreover, fat load varies considerably, only one recent review by an expert panel recommended a preferable amount of fat of about 75 g [66].

This is the first comparative study using a broader spectrum of tests in one and the same patient. Platelet function tests show a great variability and differ between the groups examined. As no single standardized and validated platelet function test is available, no clinically valid conclusions can be drawn.

## Abbreviations

ADP: Adenosin diphosphate; DM: Diabetes mellitus; HC: Hypercholesterolemics; HDL: High-density lipoprotein; HTG: Hypertriglyceridemia; MS: Metabolic syndrome; PF4: Platelet factor 4; PFA: Platelet function analyzer; PRP: Platelet rich plasma; PFA-100: Platelet function analyzer; PF: Platelet sensitivity; PG: Prostaglandins; TG: Triglycerides; ßTG: ß-thromboglobulin; TX: Thromboxane; VLDL: Very low-density lipoproteins.

## Competing interests

The authors declare that they have no competing interests.

## Authors' contributions

HS and RB reviewed the literature, drafted the manuscript, and revised the manuscript. All authors read and approved the final manuscript.
